# Alterations in the gastric microbiota and metabolites in gastric cancer: An update review

**DOI:** 10.3389/fonc.2022.960281

**Published:** 2022-08-23

**Authors:** Changzhen Lei, Daojun Gong, Bo Zhuang, Zhiwei Zhang

**Affiliations:** Department of Gastrointestinal Surgery, Jinhua Hospital of Zhejiang University, Jinhua, China

**Keywords:** gastric cancer, microbiota, metabolite, metabolomics, association analysis

## Abstract

Gastric cancer (GC) is one of the leading causes of cancer mortality worldwide. Numerous studies have shown that the gastric microbiota can contribute to the occurrence and development of GC by generating harmful microbial metabolites, suggesting the possibility of discovering biomarkers. Metabolomics has emerged as an advanced promising analytical method for the analysis of microbiota-derived metabolites, which have greatly accelerated our understanding of host-microbiota metabolic interactions in GC. In this review, we briefly compiled recent research progress on the changes of gastric microbiota and its metabolites associated with GC. And we further explored the application of metabolomics and gastric microbiome association analysis in the diagnosis, prevention and treatment of GC.

## Introduction

Gastric cancer (GC) is the fourth most common cancer in the world, and its cancer-related mortality rate ranks second in the world (1). According to statistics, GC was responsible for over 1,089,103 new cases and 768,793 deaths in 2020 (2). Currently, there is no effective treatment for the disease, and the lag in the diagnosis of early GC is a major cause of high mortality in cancer patients. Endoscopy is now widely used for early screening, but not only does this method involve invasive procedures, its accuracy depends on the experience of the endoscopist and pathologist, and its economics are still questionable (3). Therefore, it is of great significance to study non-invasive and specific biomarkers for early screening, diagnosis and treatment of GC.

The main risk factors of GC include *Helicobacter pylori* infection, smoking, dietary factors, etc (4). *H. pylori* infection is widely recognized as a high risk factor for the development of GC (5). In many developing countries, the *H. pylori* infection rate exceeds 90% (6). Almost all cases of GC are associated with *H. pylori* (7). It was previously believed that the highly acidic environment in the stomach is not suitable for bacterial growth in addition to *H. pylori.* However, with the advancement of sequencing technology, it has proven that the stomach is inhabited by a robust microbiota (4) and changes in gastric microbes may be a possible cause of GC. Studies have found that microbial diversity is significantly lower in GC patients compared with those with superficial gastritis (8, 9) and GC is associated with increased microbial diversity and richness (10, 11).

Microbial metabolites have been shown to play important roles in cancer initiation or progression (12–14). In recent years, with the the development of metabolomics technology, metabolomics has been used to characterize the metabolic perturbation and identify potential biomarkers in various cancers (15). Combining metagenomics and metabolomics may further help us understand the relationship of microbial dysbiosis and harmful metabolites to gastric carcinogenesis, providing new ideas for us to understand the mechanism of GC (16). This review summarizes the research progress on GC-related alterations in gut microbiota and its metabolites, the application of metabolomics and gut microbiome in the diagnosis, prevention, and treatment of GC, and discusses current challenges and future directions.

## Microbiome: an overview

The human body is colonized with a large and complex microbial community, and the sum of its genes is called the human microbiome. The performance of the human microbiome is influenced by a variety of environmental and physiological changes, including age, gender, diet, and more. Studies (17–19) found human microorganisms are closely related to a variety of diseases such as infectious diseases, obesity, diabetes, liver disease, coronary heart disease and tumors, etc. They form a symbiotic relationship with the host during the coevolution process, and play an important role in regulating the host’s digestion, absorption, metabolism, and immunity (20, 21). Among them, the human digestive tract is a huge microbial reservoir, and the total number of cells is 10 times that of the total number of human cells, and the number of genes contained in it is 150 times that of the total human genome (22). The digestive tract contains a large spectrum of pathogenic commensal bacteria. These microorganisms exist in the gut in a symbiotic form in a healthy state, and maintain the health of the body together with the host; in an unhealthy state, their diversity and abundance change abnormally, leading to the occurrence and development of various diseases, such as esophageal reflux, gastritis, pseudomembranous colitis (23) and other digestive tract diseases, and tumor diseases such as liver cancer (24) and gastric cancer (25). Therefore, the gastrointestinal microbiota is considered as a potential therapeutic target for various disease interventions. For example, studies have shown that fecal transplantation is significantly effective in improving symptoms of recurrent diarrhea due to *Clostridium difficile* infection (23). At present, the research on the mechanism of human microorganisms in the occurrence and development of various diseases is still in its infancy. Although the international research on the relationship between human microecology and diseases is becoming more and more intense, there are many key technologies and problems in the field of human microecology research that need to be further explored. Herein, we reviewed the research progress on the relationship between gastrointestinal microecology and GC. In **Table 1** we summarize the changes in microbial diversity in gastric cancer.

**Table 1:** Summary of studies examining the changes in microbial diversity in gastric cancer.

Sample size	Method	Variable region	Major findings	Ref.
SG (n=21), AG (n=23), IM (n=17), GC (n=20)	16S rRNA gene sequenceing *via* Mothur software	V4	Twenty-one bacterial groups, including *Peptostreptococcus, Streptococcus anginosus, Slackia, Gemella* and *Fusobacterium* were enriched in GC. Ten bacterial groups including *Vogesella, Candidatus Portiera, Comamonadaceae* and *Acinetobacter* were decreased in GC. Oral microbes *P. stomatis, S. exigua, P. micra, S. anginosus* and *D. Streptococcus pneumoniae* may play a key role in gastric carcinogenesis.	(8)
GC (n=48), Control group(n=120)	16S rRNA gene sequenceing *via* Illumina MiSeq	V3-V4	*Lactobacilli* and *Enterococci* were the dominant genus in several cancer patients. C*arnobacterium, Glutamicibacter, Paeniglutamicibacter, Fusobacterium and Parvimonas* were associated with GC.	(9)
GC (n=12), FD (n=20)	16S rRNA gene sequenceing *via* Illumina MiSeq		Several bacterial taxa are enriched in the GC, including *Lactococcus, Veillonella* and *Fusobacteriaceae* (*Fusobacter* and *Leptinobacterium*).	(10)
GC (n=54), CSG (n=81)	Next Generation Sequencing	V5-V6	In GC, microbial diversity and the abundance of *H. pylori* was significantly decreased, and the abundance of intestinal commensals increased. Bacteria with functions of nitrate reductase and nitrite reductase increased.	(11)
HC (n=30), CG (n=21), IM (n=27), IN (n=25), GC (n=29)	16S rRNA gene sequenceing *via QIIME* 1.9.1	V4	The bacterial diversity and abundance of *Armatimonadetes, Chloroflexi, Elusimicrobia, Nitrospirae, Planctomycetes, Verrucomicrobia* and *WS3* phyla decreased gradually from CG, IM, IN to GC. *Actinobacteria, Bacteroidetes, Firmicutes, Fusobacterium, SR1* and *TM7* were enriched in IN and GC.	(26)
CG (n=9), IM (n=7), GC (n=11)	16S rRNA gene sequenceing *via* Illumina MiSeq	V3-V4	The frequency and abundance of *H. pylori* were significantly lower in the cancer group. *Clostridium, Fusobacterium* and *Lactobacillus* are enriched in GC, *Clostridium colon* and *Fusobacterium nucleatum* have certain diagnostic ability for GC.	(27)
CG (n=6), GC (n=6)	16S rRNA pyrosequencing	V1-V3	The bacterial load in GC was significantly increased. The microbiota composition of GC was not significantly different from that of CG. *Lactobacillus, Escherichia-Shigella, Nitrospira, the fungus Burkholderia* and *uncultivated Lachnospiraceae* were enriched in GC.	(28)

SG, superficial gastritis; AG, Atrophic gastritis; IM, intestinal metaplasia; GC, gastric cancer; FD, functional dyspepsia; CSG, Chronic superficial gastritis; HC, healthy control; CG, chronic gastritis; IN, intraepithelial neoplasia.

## Methods for studying the microbiota

The traditional method for detecting gastrointestinal microbiota is mainly to quantitatively analyze the microflora by counting the number of viable bacterial clones in gastric mucosa, gastric juice, and intestinal contents. In recent years, with the development of molecular microecology research and the application of related technologies, the detection level of gastrointestinal microecological microflora has been significantly improved. The main molecular technique for studying microbiota expression is DNA amplification of hypervariable regions using a polymerase chain reaction (PCR) (29). Next generation sequencing (NGS) can realize large-scale parallel sequencing of multiple genes and can fundamentally solve the practical problems of difficult diagnosis caused by the heterogeneity of single-gene inheritance, multiple genes, and complex phenotypes. NGS advances the study of the human microbiome and helps understand the association between microbiome imbalances and disease phenotypes (29).

## Diet and GC

Diet is thought to influence the development or progression of GC, possibly through complex metabolic and immune pathways. Recent microbiome studies suggest that dysbiosis of the microbiota may be a key risk factor for the development of GC (25). High-fat dietary components, like meat and snacks, have been found to increase the abundance of bile-tolerant microbes and decrease metabolizing plant polysaccharides bacterial levels, which may induce gastrointestinal carcinogenesis (30, 31). Consuming preserved foods can lead to high salt intake, which directly damages the stomach lining and increases the formation of nitroso compounds that significantly increase the risk of GC (32). At the same time, a high-salt diet can increase the risk of *H. pylori* infection, and the synergistic effect of the two pathogens can further increase the risk of GC occurrence and development (33). A study showed that a healthy dietary pattern characterized by the consumption of vegetables, fruits may reduce the risk of GC (34). Fresh vegetables contain various types of antioxidants that act as protective agents, potentially ameliorating the effects of microbial imbalances. Several antioxidant-related nutrients present in fruits also play a key role in the prevention of GC (35, 36). Dairy products containing probiotics may reduce the risk of various types of gastrointestinal cancers by modulating immune parameters (37). It reduces the levels of several cancer-related biomarkers resulting from microbial and metabolic imbalances, while increasing the production of IFN-γ, which has anticancer effects (38). Therefore, dairy products, fresh vegetables and fruits should be included in the daily diet to reduce the risk of GC.

## The Mechanisms of GC mediated by gastrointestinal microbiota

A dysbiosis of the microbiota occurs when the composition of bacterial species and the number of harmful bacteria changes, thereby promoting the development of GC (25). At present, the complex micro-ecosystem in the stomach has attracted the attention of many researchers. Bik (39) et al. found through gene sequencing that there are 128 phylotypes in the gastric microflora, belonging to 8 phyla, of which the dominant microflora accounts for 5 phyla, namely *Bacteroidetes, Firmicutes, Fusobacterium*, and *Nephronomyces and Proteobacteria*. *H. pylori* can colonize the human gastric mucosa, leading to dysbiosis in the gastrointestinal tract, resulting in chronic active gastritis. It can further cause peptic ulcer or malignant gastric epithelial mucosal lesions, affecting the immune function of gastric mucosal epithelial cells (40, 41). Maldonado-Contreras A (42) et al. analyzed the gastric microflora of *H. pylori*-positive patients and showed that *H. pylori* infection increased *Proteobacteria, Helicobacter* and *Acidobacteria*, while reducing *Actinomyces, Bacteroidetes* and *Firmicutes*, significantly changing the bacterial abundance in the stomach. Wang (26) et al. found that the abundance of *Armatmonadetes, Chloroflexi, Elusimicrobia, Nitrospirae, Planctomycetes, Verrucomicrobia*, and *WS3* decreased sequentially from CG, IM, IN to GC. *Actinobacteria, Bacteroides, Fusobacterium, SR1* and *TM7* were more abundant in IN and GC. At the community level, the proportions of Gram-positive and anaerobic bacteria were higher in IN and GC than in other tissue types, while the proportions of aerobic and facultative anaerobes were significantly reduced in GC, suggesting that these bacteria may play a role in the process of intestinal metaplasia and GC. Below we discuss some of the microbiota associated with GC.

*H. pylori* is a Gram-negative, microaerophilic pathogen that colonizes the human gastric mucosa, and is closely related to the occurrence of chronic gastritis, peptic ulcer, and GC (43). The World Health Organization has classified it as a class I carcinogen Factors (44). *H. pylori* is involved in the occurrence and development of GC through pathogenic virulence factors such as vacuolar toxin-associated protein A (VacA), cytotoxin-associated protein (CagA), urease, adhesion factor, blood group antigen-binding adhesin gene, lipopolysaccharide, and inflammation and immune response after infection, etc (45). Lertpiriyapong (46) et al. studied the *H. pylori* INS-GAS mouse model and found that IL-11, TGF-β and cancer-related gene expression increased, which promote the development of GC. Hayashi (47) et al. showed that CagA secreted by *H. pylori* can up-regulate the expression of c-MYC, DNMT3B, EZH2, down-regulate the expression of miR-26a and miR-101, and decrease the expression of let-7, thereby activating the Ras pathway and increasing Ras expression, thus participating in the occurrence and development of GC. Cheng (48) et al. applied genome-wide methylation analysis and found that, In human *H. pylori*-related GC tissues, *H. pylori* can induce the methylation of FoxD3 and inhibit the activation of cell death regulators CYFIP2 and RARB, thereby promoting the proliferation and invasion of GC cells.

*Enterococcus faecalis* induce intracellular production of oxidative phosphorylation-independent ROS while disrupting the mitochondrial genome in gastric cells. The bacteria also induce a pro-inflammatory response driven by NF-κB, one of the key transcription factors in cancer-related inflammation. It also impairs the DNA damage response and cell cycle-controlled gene expression, induces mitochondrial DNA instability, and promotes tumor formation. *E. faecalis* infection also resulted in decreased expression of several genes involved in DNA repair, such as MMR gene expression (49).

*Escherichia coli* can release cytotoxins such as colibacins, which cause DNA damage (50). At the same time, it can also induce inflammation, destroy gastrointestinal mucosal cells, and promote the production of GC (51).

*Fusobacterium nucleatum* is one of the enriched strains in the GC microbiota. It can directly act on host cells and affect the expression of cancer marker genes, thereby promoting the occurrence of cancer (52). In addition, *Fusobacterium nucleatum* can also secrete endotoxin to inhibit the immune function of the body and generate an inflammatory microenvironment (53). Hsieh (27) et al. studied the bacterial species associated with gastric epithelium in 11 GC patients and found that *Fusobacterium nucleatum* was abundantly enriched in GC patients, and the gastric microbes of most GC patients were different from those of noncancerous gastric disease patients. The experimenter operating characteristic curve analysis showed that the sensitivity of *Fusobacterium nucleatum* combined with *Clostridium colicanis* and *Fusobacterium canifelinum* in the diagnosis of GC was 100%, and the specificity was about 70%. This suggests that *Fusobacterium nucleatum* may be highly correlated with GC. In addition, Abed (54) et al.found that Gal-GalNAc antigen was highly expressed in GC tissue. *Fusobacterium nucleatum*, as a cancer-promoting biological factor, can be enriched to the lesion through the specific interaction of Fap2 with Gal-GalNAc antigen on the tumor surface, and lead to the expression of MUC2 and TNF-α in cancer cells (55). According to the above studies, it is speculated that *Fusobacterium nucleatum* may be involved in the development of GC and can be used as a potential biomarker for monitoring GC.

A significant increase in the relative abundance of lactic acid-producing bacteria (*Lactococcus* and *Lactobacillus*) was observed in GC patients (10). While Lactobacillus species are commonly used as probiotics and are thought to be beneficial to the host, elevated lactate levels can be very harmful in the context of cancer. Lactic acid can act as an energy source for tumor cells, inducing glycolysis leads to increased ATP supply, also can promote inflammation and stimulate tumor angiogenesis. In addition, Lertpiriyapong (46) et al. found that *Lactobacillus* can accelerate the development of *H. pylori*-associated gastritis into intraepithelial neoplasia in the *H. pylori* INS-GAS mouse model.

Wang (28) et al. reported that the phylum *Nitrospirae* was present in all patients with GC but completely absent in patients with chronic gastritis. Notably, several members of the *Nitrospirae* phylum are known to play a role in the metabolism of nitrates and nitrites (56). It is known that the consumption of nitrates is a significant risk factor for the development of GC, and it is plausible that these bacteria may increase cancer risk.

At present, the application of probiotics and their metabolites in the treatment of gastrointestinal diseases has attracted more and more attention. Probiotics are a class of active microorganisms that are beneficial to the host. They colonize the human gastrointestinal tract and reproductive system, and can produce exact health effects and improve the host’s micro-ecological balance. Studies (57, 58) have shown that probiotics and their metabolites have inhibitory effects on *H. pylori*, which can inhibit the colonization and growth of *H. pylori* in gastric mucosal epithelium, reduce H. pylori activity, and kill *H. pylori* by destroying the cell wall. Fermented milk formed by the fermentation of *Propionibacterium freudenreichii* can promote the apoptosis of human GC cell line HGT-1, and can induce typical apoptosis processes, including chromosome aggregation, apoptotic body formation, and cell apoptosis, et al. And the fermented milk can enhance the cytotoxicity of the GC chemotherapeutic drug camptothecin. *Lactobacillus casei* extract can inhibit the proliferation of GC cell line KAT03 and induce its apoptosis by inactivating the NF-κB promoter activity. Further molecular mechanism study found that *Lactobacillus casei* extract can reduce the expression of NF-κB and I-κB, and then some molecules in the mTOR signaling pathway such as PI3K, Akt and p70S6 kinase phosphorylation decrease, which promotes the occurrence of apoptosis (59). A study by Orlando (60) et al. found that the cytoplasmic extract of *Lactobacillus* rhamnosus strain GG could significantly inhibit the proliferation of GC cell HGC-27 strain and colon cancer cell DLD-1 strain, indicating that *Lactobacillus* mainly relies on cytoplasm to exert its anti-tumor proliferation effect. Mahkonen (61) et al. applied *Lactobacillus* and *Bifidobacterium* to stimulate primary human GC cells AGS and metastatic human GC cells NCI-N87, and detected cyclooxygenase-1 (COX-1), COX-2, COX-1-IR expression. The results showed that the expression of COX-1 increased after *Lactobacillus* stimulated NCI-N87 cells, while the expression of COX-1, COX-2 and COX-1-IR did not change significantly after *Bifidobacterium* stimulated AGS and NCI-N87 cells, suggesting that *Lactobacillus* can inhibit the growth of metastatic GC cells by inducing the production of cytoprotective COX-1.

## Metabolism and GC

Gastrointestinal microbes play an important role in human health and disease. The metabolic functions of gastrointestinal microbes can be considered as contributing factors for disease development, and their bioactive substances have important effects on the physiological and pathological processes of the host. With the rise of metabolomics, significant progress has been made in understanding the relationship between metabolic regulation and cancer. Extensive studies have shown that metabolic disturbances are one of the hallmarks of cancer (62) and are intricately linked to tumorigenesis and cancer immune escape (20). To understand this connection, We must first understand the metabolic changes in GC and the mechanisms behind these changes (63). In **Table 2** we summarize the altered metabolites in different sample in GC.

**Table 2:** Metabolic changes in gastric cancer.

Sample type	Sample size	Analytical method	Multivariate method	Major findings	Ref.
Urinary (Male SCID mice SGC-7901 cell line)	GC (n=16) (metastasis group =8 and non-metastasis =8) Control group (n=8)	GC-MS	PCA	Decreased levels of alanine, glycerol, L-proline, butyric acid, and L-threonine and elevated levels of succinic acid and inositol can predict GC metastasis.	(64)
Urinary	GC (n=112), HC (n = 87)	GC-MS	OPLS-DA	Alanine, glycine, valine, isoleucine, serine, threonine, proline, methionine, tyrosine, tryptophan, ethyl 2-methylacetoacetate, levulinic acid, benzlmalonic acid and p-cresol can be used as candidate biomarkers for clinical GC diagnosis. Proline, p-cresol and 4-hydroxybenzoic acid can predict the patient′ prognosis.	(65)
Tissue	human GC subjects (n=125) and normal controls ( =54)	^1^H NMR	OPLS-DA	Isoleucine, lactate, glutamate, glutathione, TMAO, 4-hydroxyphenylactate, tyrosine, phenyacetylglutamine, hypoxanthine, citrulline, valine, acetoacetate and methylamine are changed along with the development of GC. These modified metabolites revealed disturbances in glycolysis, glutaminolysis, TCA, amino acid, and choline metabolism, which are associated with the development and progression of GC.	(66)
Plasma	human GC subjects (n=84) and GU (n=82)	LC-MS/MS	PLS-DA	Glutamine, ornithine, histidine, arginine and tryptophan, was identified for discriminating GC and GU with good specificity and sensitivity.	(67)
Plasma	80 patients (19 NAG−, 20 CAG+, 21 PLGC and 20 GC)	UPLC-MS/MS	PCA/PLS-DA	Tryptophan and nitrogen metabolism pathways are significantly altered. Tryptophan, phenylacetylglutamine and histidine can distinguish the non-GC group from the GC group.	(68)
Tissue (Male SCID mice SGC-7901 cell line)	GC (n=16) (metastasis group =8 and non-metastasis =8) Control group (n=6)	GC-MS	PCA	Proline was the most increased tissue metabolite in the metastatic group, and compared with the non-metastatic group, its expression increased 2.45-fold. Proline metabolisms plays an important role in GC metastasis.	(69)
Plasma	human GC subjects (n=30) and normal controls (n=30)	GC-MS	OPLS-DA	Metabolites such as valine, sarcosine, adipic acid, and cholesterol may be potential biomarkers for clinical GC diagnosis.	(70)
gastric juice	CSG (n=20), IM (n=12) and GC(n=38)	LC-MS/MS	PCA	Bile acid imbalance may be directly associated with GC and indirectly influence stomach carcinogenesis *via* overexpression of histidine decarboxylase.	(71)

SCID, severe combined immune deficiency; GC-MS, gas chromatography-mass spectrometry (1);H NMR (1),hydrogen-nuclear magnetic resonance; GU, gastric ulcer; LC-MS/MS, liquid chromatography/tandem mass spectrometry; NAG−, non-active gastritis without H. pylori infection, CAG+: chronic active gastritis with H. pylori infection, PLGC, precursor lesions of gastric cancer; UPLC-MS/MS, ultra performance liquid chromatography/tandem mass spectrometry.

## Glucose metabolism

The “Warburg effect” was first proposed in 1956, and subsequent studies have shown that, compared with control group, the concentration of lactate continued to increase in GC group (64–66), and glucose was significantly depleted, proving that cancer cells increase glucose uptake and obtain energy through glycolysis to meet the energy requirements for maintaining their rapid growth and proliferation (66). At the same time, lactic acid is the final product of glycolysis, and accumulated lactate modulates the activity of proteases that break down the extracellular matrix. These proteases can produce peptides and amino acids that can be used for energy production (72). The acidotic microenvironment also contributes to the formation of cancer blood vessels to meet the nutrients required for tumor invasion and metastasis (73). Based on metabolomic studies, it was found that the level of glucose involved in glycolysis decreased in both the tissues and plasma of GC patients and SGC-7901 tumor-bearing mice, so the glycolytic state may be of great significance for the early diagnosis of GC (66, 74, 75). Studies have reported that in non-diabetic conditions, blood glucose levels are positively correlated with cancer mortality, and the potential mortality of cancer patients with glucose intolerance is also increased (76). Hyperglycemia in patients with both diabetes and GC can promote the proliferation of GC cells and reduce the sensitivity to chemotherapeutic drugs (77). Not only that, glycolysis transcriptional regulators and glycolysis-related proteins are significantly correlated with the prognosis of cancer patients, so glycolysis status may also be a potential biomarker for prognosis (78).

## Amino acid metabolism

The tumor microenvironment is far from an ideal cell growth environment, and nutrients such as amino acids can be used as energy sources for tumors to affect the occurrence and development of tumors (79). Many cancer cell lines cannot survive without glutamine (80). Glutamine is produced by fermentation of glutamate-producing bacteria, and it is required for anabolic growth of mammalian cells because of its ability to control protein translation (81). Moreover, reprogramming of glutamine metabolism further promotes proliferative and metabolic responses regulated by the oncogenic transcription factor c-MYC (16, 82). In addition, studies (67, 68) have found that patients with GC have lower levels of tryptophan, which may be due to up-regulation of the expression of tryptophan metabolizing enzymes indoleamine 2,3-dioxygenase 2 and tryptophan dioxygenase (68). This alteration not only promotes cancer progression, but also affects tumor immune regulation. Chen (69) et al. used the human GC cell line SGC-7901 to establish a metastatic and non-metastatic animal model of GC. They found that proline was the most increased tissue metabolite in the metastatic group, and compared with the non-metastatic group, its expression increased 2.45-fold. Elevated proline may be due to the degradation of extracellular matrix and collagen in the microenvironment (83). And they point out that proline metabolism may play an important role in metastasis. However, further functional and clinical sample analysis of the metabolic pathways is needed to demonstrate their role in GC metastasis.

## Lipid metabolism

Gut microbes regulate dietary lipid composition, digestion, and absorption, potentially altering intestinal lipoprotein formation. Lipids mainly include fats, phospholipids and sterol. Disorders of lipid metabolism may affect the proliferation and differentiation of tumor cells and accelerate the occurrence and development of cancer70. Song et al. (84) found that serum cholesterol and some fatty acid levels in patients with GC were significantly reduced. It is suggested that GC cells may consume a large amount of fatty acids to meet the needs of cell membrane synthesis and energy production. The survival of cancer cells in the human body depends on lipids, and accumulated lipid droplets are found in various cancer microenvironments (70), so lipid droplets are expected as effective targets for blocking tumor growth (85), and fatty acid metabolism-related proteins may also become diagnostic markers for early GC (86, 87). In addition, GC is prone to omental metastasis, and fatty acid oxidation is regulated by omental adipocytes. However, fatty acid oxidation is enhanced in GC patients, which promotes omental metastasis of GC (88, 89). Fatty acid oxidation also plays an important role in mesenchymal stem cell-mediated chemoresistance in patients with GC (90). Study has shown that fatty acid oxidation inhibitors combined with chemotherapeutic drugs can improve the chemoresistance of patients (91). Notably, the level of O-acetylcarnitine, which increases fatty acid β-oxidation, tends to decrease as early GC progresses to advanced stages (92). This seems to explain the significant reductions in 9-hexadecenoic acid, cis-vaccenic acid, arachidonic acid, hexadecanoic acid and 3-hydroxybutanoic acid in stage III/IV GC tissue samples (70). However, this difference needs to be further elucidated with larger samples and different analytical methods. In addition, patients with advanced GC are often accompanied by cachexia. Study has found that chronic inflammation mediated by TNF and IL-6 may promote the occurrence of cachexia in patients with GC. In the early stage of cachexia, serum TNF is positively correlated with serum free fatty acid (FFA). In early and advanced cancer cachexia, serum IL6 and FFA were also significantly positively correlated (90), indicating that cachexia may be associated with lipid metabolism, but the specific mechanism is still unclear.

## Nucleotide metabolism

The rapid proliferation and differentiation of tumor cells can lead to abnormal nucleotide synthesis and catabolism. Nucleotides are related to energy metabolism, mainly in the form of ATP and GTP. At the same time, nucleotide synthesis can ensure the timely replication of DNA. These are all necessary for tumor cell proliferation and key elements of cancer metabolism (16, 93, 94). Studies (75, 84, 95) have found that compounds involved in nucleotide metabolism, such as adenine, xanthine, and inosine are increased in GC patients or animal models, which can be used as biomarkers for early diagnosis of GC. Nucleotide catabolism is characterized by hyperuric acid or hyperurate in GC patients (64, 96). Some purine compounds, such as hypoxanthine and guanosine, increased the accumulation of urea and uric acid in the urine of GC patients, further indicating abnormal nucleotide metabolism in GC patients (97, 98). In addition, nucleotide-related proteins, such as nicotinamide nucleotide transhydrogenase, are also involved in the growth and metastasis of GC (99).

### Other Metabolism

Bile acids are products of cholesterol catabolism in the liver. Bile acids are known to be involved in the pathological mechanism of gastric carcinogenesis. Lee (71) et al. found that the metabolism of cholic acid to deoxycholic acid was statistically different on the basis of the progression of chronic superficial gastritis to GC, suggesting that bile acid imbalance may be directly related to GC. Bile acids are cell-surface G protein-coupled receptor 1 (TGR5) ligands that regulate intestinal barrier formation and inflammation-driven immune dysfunction. Studies have shown that TGR5 is overexpressed in gastrointestinal adenocarcinomas and is associated with poor prognosis in GC patients (100). In addition, the bile acid receptor Farnesoid X receptor (FXR) is associated with the expression of Caudal type homeobox 2 (CDX2) and Mucin 2 (MUC2), which can lead to gastrointestinal metaplasia (101). Bile acids induce upregulation of Egr-1 and oncogenes through MAPK signaling in GC cells. Egr-1 has been implicated in biological processes including inflammation, cell proliferation, cell differentiation and cancer progression (102). Primary bile acids can also increase the expression levels of c-MYC and c-Jun genes through MAPK signaling, which are involved in gastric carcinogenesis and progression (103, 104). Continued exposure of gastric epithelial cells to primary bile acids may be a factor in gastric carcinogenesis. Furthermore, studies have shown that the signaling of hydrophobic bile acids is mediated through PKC activation and COX-2 induction, which leads to increased cell invasion. Moreover, by perturbing the bile acid pool, ursodeoxycholic acid (UDCA) was able to attenuate chenodeoxycholic acid CD-induced PGE2 synthesis and tumor invasiveness without affecting COX-2 expression (105).

TMAO is formed by choline, the precursor of trimethylamine (TMA), through the combined action of gut microbes and the liver. Changes in TMAO levels are closely related to gut microbe disturbances (106). Studies have shown that urinary TMAO can be used as a predictor of GC (107).

Nitrite is a precursor of carcinogenic nitroso compounds, and studies have found that the GC microbiota has a nitrate reductase function that promotes the reduction of nitrate to nitrite (55).

## The microbiome-metabolomics interplay in GC

The possible role of gut microbiota and metabolomics in cancer prevention and treatment has received extensive attention (108). Studies (109) have found that eradication of H. pylori may play an important role in preventing the occurrence and malignant progression of GC. In addition, various probiotics are widely used in daily life, which can protect the gastric mucosa and enhance the immune response. Kim (107) et al. used (1)H NMR studies to find metabolites related to the tricarboxylic acid (TCA) cycle, such as TMAO, TMA, 3-indolyl sulfate, hippurate, citrate and 2-oxoglutarate can be considered as a therapeutic target to enhance the efficacy of ADR. Tumor Microenvironment(TME) highly affects the metabolism of cancer cells. It provides nutrients to tumor cells and prepares the surrounding environment for proliferation, local invasion and metastasis. The microbiota, through its metabolites, can shape the TME to influence tumor development (110). However, studies investigating the interaction between the microbiome and metabolome in GC are limited. Only a few reports have highlighted the association between the microbiome and metabolome in GC. Coker (8) et al. found the functional changes in the GC microbiomes included significantly increased representation of predicted KEGG (Kyoto Encyclopedia of Genes and Genomes) pathways involved in nucleotide metabolism, carbohydrate digestion and absorption and bacterial ion channels compared with other disease stages. The disturbances in the gut microbiota lead to the production of harmful metabolites, such as acetaldehyde, secondary bile acids, and glucuronic acid, which induce DNA damage and promote gastric carcinogenesis. A study (110) found that polyamines (putrescine, spermidine and spermine) play an important role in cell proliferation and are required to maintain cell growth in both pre-tumor and tumor tissues. Probiotics can affect polyamine metabolism by affecting polyamine metabolism and act as an antineoplastic agent in the stomach. For example, lyophilized and sonicated preparations of *Lactobacillus brevis* CD2 inhibit arginine-dependent polyamine synthesis, thereby inducing associated apoptosis (111). Dai (112) et al. studied the interaction between gastric microbiota and metabolites in GC, and conducted an association analysis between different genera and different categories of metabolites. They found the metabolome profiles of the GC tumor tissues were strongly influenced by *Helicobacter, Lactobacillus*, and other microorganisms, which might promote GC development, suggesting that combined analysis of microbiota metabolites with microbiota bacteria may serve as promising diagnostic biomarkers for GC.

## Conclusions

GC is one of the most malignant tumors in the world, although its pathogenesis is still unclear, but with the advent of omics studies, there have been encouraging findings. The development of GC is not only affected by the presence of intestinal microflora, but also by metabolic changes. Gastrointestinal microflora, especially some special bacteria, affect the development of GC through metabolic and structural changes, which can provide research data for establishing preventive strategies for GC and determining its pathogenic mechanism. Although metabolomics has been able to explain the biology of GC to a certain extent, it is easily affected by background interference during the detection process, and sometimes cannot fully explain the biological process. Therefore, the comprehensive application of multi-omics methods is required (113). However, studies on the correlation between the microbiome and metabolome in GC are still very limited. Therefore, large-scale studies, including retrospective and prospective studies, are still needed in the future to reveal the impact of the microbiome and metabolomics in the GC microenvironment and how their complex interactions affect the occurrence and development of tumors, and to screen out GC diagnostic markers with high sensitivity and specificity for the diagnosis and prognosis of GC.

## Author contributions

LCZ, ZB, ZZW: Manuscript writing, literature research, editing the manuscript. GDJ: Manuscript writing and final approval of manuscript. All authors have read and approved the manuscript.

## Funding

The study was supported by grants from the Foundation of the Science and Technology Department of Jinhua (No 2021-3-066) and Jinhua Municipal Central Hospital Foundation (No JY2020-5-03).

## Conflict of interest

The authors declare that the research was conducted in the absence of any commercial or financial relationships that could be construed as a potential conflict of interest.

## Publisher’s note

All claims expressed in this article are solely those of the authors and do not necessarily represent those of their affiliated organizations, or those of the publisher, the editors and the reviewers. Any product that may be evaluated in this article, or claim that may be made by its manufacturer, is not guaranteed or endorsed by the publisher.
